# Mechanochemical Solvent-Free and Catalyst-Free One-Pot Synthesis of Pyrano[2,3-d]Pyrimidine-2,4(1H,3H)-Diones with Quantitative Yields

**DOI:** 10.3390/molecules14010474

**Published:** 2009-01-19

**Authors:** Sara Mashkouri, M. Reza Naimi-Jamal

**Affiliations:** Organic Chemistry Research Laboratory, Department of Chemistry, Iran University of Science and Technology, 16846 Tehran, Iran

**Keywords:** Solvent-free reaction, Barbituric acid, Pyrimidinediones, Heterocyclic compounds, Ball-milling.

## Abstract

Solvent-free synthesis of pyrano[2,3-d]pyrimidine-2,4(1*H*,3*H*)-diones by ball-milling and without any catalyst is described. This method provides several advantages such as being environmentally friendly, using a simple workup procedure, and affording high yields.

## Introduction

Recently, much attention has been paid to the development of new methods for the synthesis of heterocyclic compounds, due to their potential importance in the pharmaceutical and agricultural fields. Among the others, pyrimidine derivatives are of high interest, because they generally show diverse biological properties such as antitumor, analgesic, antibacterial, and fungicidal activities [1,2,3,4].

Solvent-free reactions lead to new environmentally benign procedures that save resources and energy. These kind of reactions promise to be an essential facet of ‘Green Chemistry” and are of high interest from both the economical and synthetic point of view. Solvent-free reactions possess some advantages over traditional reactions in organic solvents, for example they not only reduce the burden of organic solvent disposal, but also enhance the rate of many organic reactions.

Multicomponent reactions (MCRs) are generally one-pot reactions, in which three or more starting materials react together to form a product, where basically all or most of the atoms contribute to the newly formed product [5]. In an MCR, a product is assembled according to a cascade of elementary chemical reactions. These kind of reactions profit from avoiding unnecessary separation and purification procedures.

Ball-milling is referred generally to be a mechanical technique which is widely applied for the grinding of minerals into fine particles, and the preparation or modification of inorganic solids [6,7,8,9]. Its applications in the organic synthesis are relatively scarce, but have attained more importance during the past decade. Kaupp *et al*. have discovered the usefulness of ball-milling in organic synthesis which has been the subject of some papers and reviews [10,11,12,13,14]. Some recent examples of using a ball-mill in organic synthesis include solvent-free Knoevenagel condensation [15], protection of diols and diamines [16], functionalization of fullerenes [17]**,** reductive benzylation of malononitrile [18]**,** preparation of phosphorus ylides [19]**,** Heck-type cross-coupling reactions [20], and enantioselective organocatalytic aldol reaction [21]. Numerous typical examples of scaling up of the use of ball-mills have been given for organic and some inorganic syntheses. These include solvent-free salt formations, complexations, condensations of amines, heterocyclic syntheses, Knoevenagel condensations, cascade reactions, halogen additions, stereo- and regiospecific protective reactions, and redox reactions [10, and references therein].

The general procedures for the preparation of pyrano[2,3-d] pyrimidine-2,4(1*H*,3*H*)-diones include the reaction of arylidenemalononitriles with barbituric acid under traditional hot reaction conditions [22,23] or under microwave irradiation [24]. In these methods the requisite arylidenemalononitriles are previously prepared from malononitrile and aldehydes. Recently, direct condensation of aldehydes, malononitrile and barbituric acid in aqueous media has been reported under ultrasound irradiation [25], or catalyzed by diammonium hydrogen phosphate [26].

**Scheme 1:**
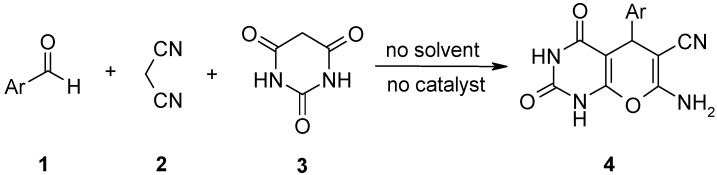
Quantitative preparation of pyrano[2,3-d]pyrimidine-2,4(1*H*,3*H*)-diones.

We now wish to report one-pot synthesis of pyrano[2,3-d]pyrimidine-2,4(1*H*,3*H*)-diones in high to excellent yields by simply ball-milling a stoichiometric mixture of an aldehyde, malononitrile, and barbituric acid without any catalyst or solvent and (Scheme 1).

## Results and Discussion

4-Chlorobenzaldehyde (**1**, Ar=4-ClC_6_H_4_), malononitrile (**2**) and barbituric acid (**3**) (1.0 mmol each) were placed in a clean 10 mL temperature controlled ball-mill vessel equipped with 2 stainless steel balls, the vessel was closed and the milling started at room temperature at a speed of 20-25 Hz. After every 10 min milling cycles, the progress of the reaction was monitored by TLC. The milling was continued for 90 minutes overall, after that TLC of the reaction mixture showed no significant reaction. Higher temperature millings were achieved by circulating hot water inside the double walled stainless steel ball mill beaker. Different temperatures were examined and it was found that using boiling water as circulant, the reaction was complete after 55 minutes. The produced water of the condensation reaction was simply removed from the crude product by heating at 80 °C under reduced pressure. Spectral data and the melting point of the product corresponded well with literature values. Other aldehydes were also examined and it was found that in all cases the boiling water temperature was enough high to complete the reaction. Because no excess of reagents was used, the products were generally obtained with no waste and no further tremendous purification processes were needed. In most cases the products were obtained in enough pure form, or a simple crystallization was enough, if it was necessary. The results are summarized in Table 1. The products were characterized by comparison of their spectral data and melting points with those reported in literature. It is specially noteworthy that all the products show a characteristic single peak in the ^1^H-NMR spectra at about 4.2-4.8 ppm, which corresponds to the benzylic proton of the ArCH group. The more interesting signal in the ^13^C-NMR spectra is the signal of about 58-60 ppm, which belongs to the α-C to the CN group. These results have been previously reported for the same and for similar structures [15,26].

**Table 1:** Synthesis of pyrano [2,3-d] pyrimidine-2,4(1*H*,3*H*)-diones under solvent-free and catalyst-free conditions.

Entry	Ar	Product	Time	Yield	M.P. (°C) [ref]
1	C_6_H_5_	**4a**	70	>99^a^	206-209 (208-210) [24]
2	2-ClC_6_H_4_	**4b**	90	>99^ a^	213-215 (213-215) [24]
3	4-ClC_6_H_4_	**4c**	55	>99^ a^	234-237 (239-240) [25]
4	2-NO_2_C_6_H_4_	**4d**	60	>99^ a^	257-258 (262-263) [22]
5	3-NO_2_C_6_H_4_	**4e**	15	>99^ a^	255-257 (262-263) [25]
6	4-NO_2_C_6_H_4_	**4f**	25	>99^ a^	227-229 (237-238) [25]
7	4-BrC_6_H_4_	**4g**	60	94^ b^	229-230 (230-231) [26]
8	4-CH_3_OC_6_H_4_	**4h**	30	>99^ a^	287-288 (280-281) [24]

^a^ Yields refer to conversion yields. ^b^ isolated yield

**Scheme 2:**
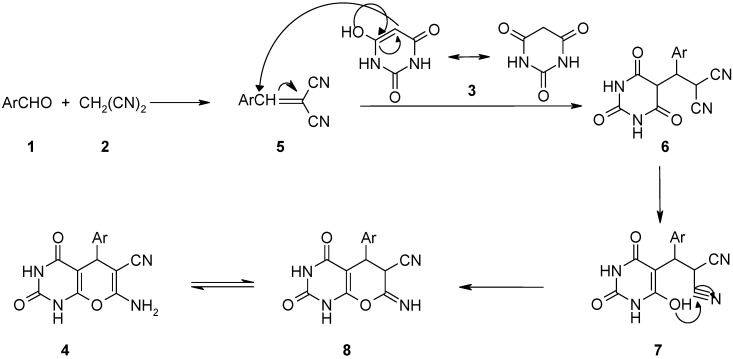
Possible mechanism for the preparation of **4**.

A possible mechanism is outlined in Scheme 2. The reaction may proceed at first via a Knoevenagel condensation of aldehyde **1** with malononitrile **2** to afford the Michael acceptor **5**. This was confirmed by the fact that at 50 °C, the reaction of 4-chlorobenzaldehzde, malononitrile, and barbituric acid produced 4-chlorobenzylidene malononitrile (**5**, Ar=4-ClC_6_H_4_), while barbituric acid was remained unchanged. Such solvent-free Knoevenagel reactions have been previously reported [15]. The active methylene of barbituric acid **3** reacted then via its enol form with **5** in a Michael addition reaction to give the intermediate **6,** which is then tautomerized to **7**. Intramolecular cyclizative condensation of **7** gave **8.** Finally the tautomerization of **8** afforded the expected products **4**.

In summary, we have reported herein a quantitative synthesis of various pyrano[2,3-d]pyrimidine-2,4(1*H*,3*H*)-diones from stoichiometric mixtures of pure reactants in a simple mechanochemical method without the necessity for use of solvents, addition or removal of catalysts, or solvent-consuming workups. The fact of no catalyst of any kind may seem remarkable at first glance, but it has been proven in recent years that many of the reactions studied, can indeed be performed done without any auxiliary [11,12,14,15,27,28].

## Experimental

### General

Melting points were determined with a Kofler hot stage melting point apparatus and are uncorrected. Ball mill apparatus was a Retch MM2000. Infrared (IR) spectra were recorded with a Shimadzu 8400s FT-IR spectrometer using potassium bromide pellets. ^1^H-NMR and ^13^C-NMR spectra were recorded on a 500MHz DRX-500 Avance Bruker spectrometer in DMSO-(d_6_). Reagents were obtained from commercial resources and were used without further purification. All products are known compounds and were identified by comparing of their physical and spectra data with those reported in the literature.

### General synthetic procedure

Aldehyde (**1**), barbituric acid **(2**) and malononitrile (**3**) (1.0 mmol each) were poured in a 10 mL stainless steel double-walled ball mill beaker equipped with fittings for circulating water. Two stainless steel balls of 12 mm in diameter were used. Ball milling was performed at 20–25 Hz frequency at 96°C temperature (using boiling water as circulant) for the time given in Table 1. The solid powder was dried at 80 °C in vacuum to give the product **4,** which was purified by recrystallization from DMF-ethanol, if necessary.
